# Supplementary material for: The Recruitment of the Recalcitrant-Seeded Cryptocarya alba (Mol.) Looser, Established via Direct Seeding Is Mainly Affected by the Seed Source and Forest Cover
Source: Plants (Basel). 2022 Oct 29;11(21):2918. doi: 10.3390/plants11212918 (PMC9658434; doi:10.3390/plants11212918)
Supplement: Supplementary file 1 [file plants-11-02918-s001.zip › plants-1871748-supplementary.pdf]

Table S1. Means per mother within seed source (plus standard errors) for germination (Germin), survival (Surv) on *C. alba* in the field experiment. Seed sources were Cuesta La Dormida (CD), Antumapu (AN), Cantillana (CA) and Cayumanque (CY).

Seed source	Mother	Germin	Surv
CD	1	12.5 (1.4)	2.5 (1.4)
	2	0.0 (0.0)	0.0 (0.0)
	3	15.8 (9.8)	8.3 (4.8)
	4	19.2 (10.2)	3.3 (1.9)
	5	5.0 (1.4)	1.7 (1.0)
	6	5.8 (5.8)	0.8 (0.5)
	7	36.7 (13.4)	0.0 (0.0)
	8	16.7 (7.4)	3.3 (1.9)
	9	1.7 (0.8)	0.0 (0.0)
	10	0.0 (0.0)	0.0 (0.0)
	11	0.0 (0.0)	0.0 (0.0)
AN	12	15 (10.4)	8.3 (4.8)
	13	13.3 (3.3)	0.0 (0.0)
	14	0.8 (0.8)	0.0 (0.0)
	15	0.8 (0.8)	0.0 (0.0)
	16	25 (12.8)	9.2 (5.3)
	17	25.8 (13)	14.2 (8.2)
	18	0.8 (0.8)	0.0 (0.0)
CA	19	59.2 (25.8)	23.3 (13.5)
	20	66.7 (8.3)	23.3 (13.5)
	21	40.8 (21.9)	17.5 (10.1)
	22	50 (16.1)	25.8 (14.9)
	23	43.3 (14.2)	16.7 (9.6)
	24	35 (8.0)	9.2 (5.3)
	25	45 (4.3)	15.8 (9.1)
	26	48.3 (6)	11.7 (6.7)
	27	55 (15.3)	19.2 (11.1)
	28	25 (10.1)	10 (5.8)
	29	27.5 (11.8)	0.8 (0.5)
	30	20 (8.7)	1.7 (1.0)
	31	25.8 (15.2)	0.0 (0.0)
	32	53.3 (26.2)	29.2 (16.8)
	33	16.7 (1.7)	5 (2.9)
	34	20 (8.8)	8.3 (4.8)
CY	35	15 (8.0)	5.8 (3.4)
	36	41.7 (9.3)	1.7 (1.0)
	37	5.8 (2.2)	0.8 (0.5)
	38	21.7 (8.2)	7.5 (4.3)
